# Application of the Ultra-Poverty Graduation Model in understanding community health volunteers’ preferences for socio-economic empowerment strategies to enhance retention: a qualitative study in Kilifi, Kenya

**DOI:** 10.1186/s12960-021-00645-5

**Published:** 2021-08-28

**Authors:** Njeri Nyanja, Nelson Nyamu, Lucy Nyaga, Sophie Chabeda, Adelaide Lusambili, Marleen Temmerman, Michaela Mantel, Anthony Ngugi

**Affiliations:** 1 Department of Family Medicine, Aga Khan University, Nairobi, Kenya; 2 Centre of Excellence for Women and Child Health, Aga Khan University, Nairobi, Kenya; 3 Kenya Medical Research Institute, Nairobi, Kenya; 4 Department of Population Health, Aga Khan University, Nairobi, Kenya; 5 Department of Obstetrics and Gynaecology, Aga Khan University, Nairobi, Kenya; 6 Department of Public Health and Primary Care, International Centre for Reproductive Health, Ghent University, Ghent, Belgium

**Keywords:** Community health volunteers (CHVs), Kenya, Socio-economic empowerment, Ultra-Poverty Graduation Model (UPG), Qualitative

## Abstract

**Background:**

A significant shortage of healthcare workforce exists globally. To achieve Universal Healthcare coverage, governments need to enhance their community-based health programmes. Community health volunteers (CHVs) are essential personnel in achieving this objective. However, their ability to earn a livelihood is compromised by the voluntary nature of their work; hence, the high attrition rates from community-based health programmes. There is an urgent need to support CHVs become economically self-reliant. We report here on the application of the Ultra-Poverty Graduation (UPG) Model to map CHVs’ preferences for socio-economic empowerment strategies that could enhance their retention in a rural area in Kenya.

**Methods:**

This study adopted an exploratory qualitative approach. Using a semi-structured questionnaire, we conducted 10 Focus Group Discussions with the CHVs and 10 Key Informant Interviews with County and Sub-county Ministry of Health and Ministry of Agriculture officials including multi-lateral stakeholders’ representatives from two sub-counties in the area. Data were audio-recorded and transcribed verbatim and transcripts analysed in NVivo. Researcher triangulation supported the first round of analysis. Findings were mapped and interpreted using a theory-driven analysis based on the six-step Ultra-Poverty Graduation Model.

**Results:**

We mapped the UPG Model’s six steps onto the results of our analyses as follows: (1) initial asset transfer of in-kind goods like poultry or livestock, mentioned by the CHVs as a necessary step; (2) weekly stipends with consumption support to stabilise consumption; (3) hands-on training on how to care for assets, start and run a business based on the assets transferred; (4) training on and facilitation for savings and financial support to build assets and instil financial discipline; (5) healthcare provision and access and finally (6) social integration. These strategies were proposed by the CHVs to enhance economic empowerment and aligned with the UPG Model.

**Conclusion:**

These results provide a user-defined approach to identify and assess strategic needs of and approaches to CHVs’ socio-economic empowerment using the UPG model. This model was useful in mapping the findings of our qualitative study and in enhancing our understanding on how these needs can be addressed in order to economically empower CHVs and enhance their retention in our setting.

**Supplementary Information:**

The online version contains supplementary material available at 10.1186/s12960-021-00645-5.

## Background

Shortage of skilled health workers in underserved areas is a key aspect of the growing human resource crisis in most low- and middle-income countries (LMICs) [1–5] and as a consequence the role of community health volunteers (CHVs) is increasingly important. CHVs are selected from their communities and trained to provide promotive, preventive and some curative health services in partnership with frontline health workers [2, 3, 6, 7].

CHVs play an integral role in improving healthcare coverage and access in LMICs. Several studies have demonstrated their effectiveness in reducing morbidity and mortality as well as improving health outcomes [8–10]. In Kenya, CHVs have been a vital part of primary health service delivery for decades bridging the communities health system gap and helping ameliorate Kenya’s shortage and inequitable distribution of health workforce [11]. Despite their vital contribution to healthcare service provision, lack of support, recognition, facilitation and incentives leads to high attrition rates in both voluntary and paid CHVs [1, 8, 11, 13]. However, higher attrition rates as associated with volunteers who often work with little or no compensation [11, 14–16]. Consequently, high attrition rates among this cadre leads to gaps in the delivery of essential services, loss of opportunity to build on expertise and increasing transactional costs arising from recurrent recruitment and training [2, 11].

The 2018 WHO guideline on CHVs recommended remuneration of practising CHVs commensurate with the job demands, complexity, number of hours, training need and their roles [17]. However, barriers to instituting these recommendations in practice remain. First, there are no standard strategies that would best support adequate incentivisation of CHVs, second, many LMIC governments do not have the funding available to provide this remuneration [17].

Factors contributing to high attrition among CHVs include a lack of support, recognition, facilitation and incentives [3, 5, 15, 16, 18–23]. However, a lack of opportunities to earn income and economic empowerment has consistently been noted as a key factor that would motivate their retention [24].

Between 2016 and 2020, Aga Khan University implemented a reproductive, maternal newborn and child health (RMNCH) project, ‘Access to Quality Care through Extending and Strengthening Health Systems’ (AQCESS) project in Kilifi County in Kenya. The project aimed to improve RMNCH outcomes for women, neonates and children under the age of five. As part of the implementation strategy, the project also aimed to strengthen CHVs activities through targeted health trainings congruent with the needs of the local communities. Previous evidence from the area showed that attrition was associated with poor support and lack of incentives [24]. On this backdrop, we conducted an exploratory study to assess challenges and preferred social economic empowerment strategies to improve CHVs retention in the area. Findings on the challenges are reported in a separate publication [25].

To understand how socio-economic empowerment strategies preferred by CHVs could be used to improve their motivation and retention, we mapped the findings of the qualitative exploration using the Ultra-Poverty Graduation Model (UPG) model. This model was selected for its effectiveness in reducing poverty, its comprehensiveness and because it is an adaptable intervention that leads to sustainable poverty alleviation beyond the donor’s period [26, 27]. When adapted to local contexts, this multifaceted approach has been used successfully, enabling beneficiaries to develop livelihoods and enhance their standard of living [28]. In this study, the UPG model assisted the researchers in understanding the CHVs views and mapping pathways for supporting their economic empowerment and ultimately retention.

## The Ultra-Poverty Graduation Model

The Ultra-Poverty Graduation (UPG) Model is a carefully sequenced internationally recognised multi-sector approach that aims to help participants to increase their income and become more economically self-reliant [26, 29]. The model emphasises on long-term investment in asset transfer, skills development, business development and resource financing as well as saving and planning for future transitions [26].

**Fig. 1:**
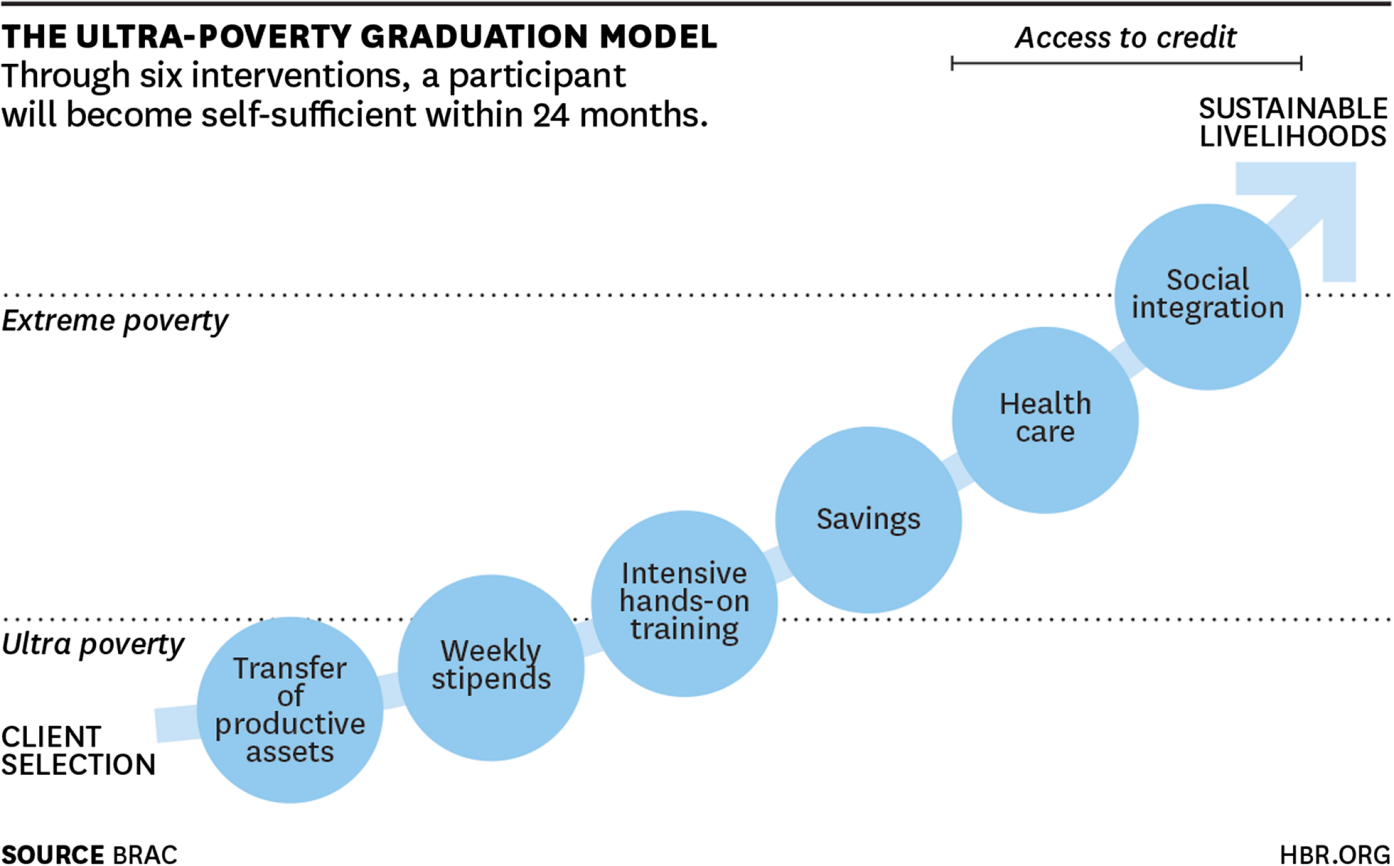
The Ultra-Poverty Graduation Model. [Reprinted with permission from "An Approach to Ending Poverty That Works" by Susan Davis Jan, January 22, 2015, hbr.org. Source: BRAC Copyright 2015 by Harvard Business Publishing; all rights reserved.]

The key aspects of the model involve a six-step intervention through which participants graduate from ultra-poverty, through extreme poverty until they achieve sustainable livelihoods over a 2-year period. Once the clients have been selected, the intervention begins with a period of consumption support involving [step 1] the transfer of assets of in-kind good such as poultry or livestock as capital and [step 2] provision of weekly stipends with consumption support to stabilise consumption. Provision of [step 3] hands-on training on how to care for assets and run a business follow and is provided alongside [step 4] savings and financial support to enhance financial literacy, build assets and instil financial discipline. Once this is sustained participants receive [step 5] healthcare provision and access and finally [step 6] social integration [26] (Fig. 1).

## Methods

### Study setting

The current study was conducted in Kaloleni and Rabai sub-counties in Kilifi county in the coast of Kenya. The two sub-counties cover an area of 909 km^2^ and have a population of about 290,000 living in about 44,000 households [30]. Children under 5 years of age comprise one-fifth of the Kilifi population and women of reproductive age account for a quarter [30]. Maternal, neonatal and child health indicators are poorer than the national averages [30, 31]. Fifty-seven percent of the population are Christian, 19% are Muslim and the remainder are traditionalists [30]. Kaloleni and Rabai sub-counties are among the poorest parts in Kenya [32]. Approximately 70% of the population lives below the poverty line [31]. Forty health facilities serve these sub-counties: 20 public/government health facilities (16 dispensaries, one health centre, one sub-district hospital, one district hospital, one military health centre), three faith-based facilities (one hospital and two dispensaries), three NGO dispensaries, and 14 privately owned dispensaries [33]. The physician-to-population ratio 10:100,000 in this area is below the national average of 19:100,000 while the nurse-to-population ratio is 40:100,000 against a national average of 166:100,000 [34]. Trained CHVs visit households for data collection, health promotion and education during their own free time, making at least one visit per month [24].

### Study design

The main study adopted an exploratory multi-method qualitative approach including Key Informant Interviews (KIIs) and Focus Group Discussions (FGDs).

### Study population and sampling methods

Study participants included CHVs and key stakeholders. These included County and Sub-county Ministry of Health and Ministry of Agriculture officials as well as multi-lateral stakeholders’ representatives from Kaloleni and Rabai sub-counties.

#### Focus group discussions

We conducted focus group discussions (FGDs) with CHVs from 10 out of the 17 Community Health Units (CHU) within the two sub-counties. The FGDs had an average of 6–10 participants sampled proportionately by gender distribution within the CHU. Participants were purposively identified by recruitment liaisons and included more experienced CHVs conversant with the Kaloleni and Rabai area.

#### Key Informant Interviews

Eight Key Informant Interviews (KIIs) were conducted with participants purposively identified as able to provide rich contextual information. Table 1 summarises the KII population.

**Table 1:** Demographics for the CHVs interviewed using focus group discussions (FGDs)

1	Gender	Total 64 female CHVs interviewed Total 17 males CHVs interviewed
2	Experience	All CHVs had worked for over a year in their station
3	Age	CHVs interviewed ranged from 26 to 67 years of age
4	Education levels	All CHVs interviewed had at least primary level of education and were literate
5	Residency	Total 81 participants interviewed; 57 were from Kaloleni and 23 were from Rabai

### Data collection process

*Interview process* To ensure the validity and reliability of the data collection tool, the principal investigator (PI) developed semi-structured questionnaires, one for the FGDs and another for the KIIs. An expert panel consisting of social scientists and an epidemiologist validated the content and construct of the semi-structured questionnaires. Originally developed in English, questionnaires were translated to Kiswahili (a national language in Kenya and the language commonly used in the Coast) and then back translated by an expert linguist. A team of research assistants underwent a 2-day training by the PI on data collection and interviewing techniques. The tools were further piloted in two FGDs using 16 CHVs who were excluded from the study. Interviews lasted between 40 and 100 min and were conducted in either English or Kiswahili based on participants’ preference. Participants in FGDs were given equal opportunity to respond to the questions as moderated by the facilitator. At the end of the interviews, the moderator and the note taker conducted debriefs and included their discussions as part of the notes.

Data collected included socio-demographic information; current income source; challenges faced while earning income; effect of CHV work on livelihood; engagement in other income-generating activities (IGAs); preference of IGAs and proposed sponsors or supporters of these engagements. For the KIIs, information was collected on; their role in engaging CHVs; challenges they perceived attributed to CHVs attrition; their sentiments on financial remuneration of CHVs; awareness of IGAs; policies in place for sustainability of IGAs and identification of key players within their institution; access to support and any ongoing or previous IGAs (Additional file 1: Appendices 1–3).

### Data management and analysis

The qualitative analyses and findings of the main study are published elsewhere [25]. These findings were mapped and contextualised using UPG model, which highlighted important factors that could be considered in the implementation of the preferred IGAs.

## Results

Total 81 participants aged between 26 and 67 years were included in the study. All participants had attained primary level education or higher and had over one year’s experience in their station. The study participants are categorised based on the interview techniques. Tables 1 and 2 provide some additional descriptions about them.

**Table 2:** Characteristics of participants interviewed using Key Informant Interviews (KIIs)

Participant	Role	Number
Ministry of Health Sub-County officials	Kaloleni Sub-County—Ministry of Health official	KII 01
Ministry of Health Sub-County officials	Rabai Sub-County Public health officer—also was representing county as a health promotion officer	KII 02
Ministry of Agriculture—Sub-County	Rabai—Agriculture extension officer—Sampled based on his experience in the community extension to provide insights on agriculture-based IGAs feasible	KII 03
IGA Trainer—Civil Society Organization (CSO)	Rabai and Kaloleni Entrepreneurship/IGA trainer—sampled based on his experience in training Community-Based Organizations (CBOs) allocated IGAs and entrepreneurial funds by the Government	KII 04
Stakeholder/CSO	Rabai Chairperson Upendo CBO group—sampled due to the role of leadership in the CBOs operations including IGAs	KII 05
Stakeholder/CSO	Rabai Youth CBO—CBO leader—sampled based on experience having IGA for the CBO	KII 06
Ministry of Health Sub-County official	Kaloleni Sub-County Community strategy focal Officer	KII 07
Ministry of Agriculture—Sub-County	Kaloleni—Agriculture extension officer—sampled based on his experience in the community extension to provide insights on agriculture-based IGAs feasible	KII 08

### Application of the UPG model to the study findings

In this section of the paper, we present data based on the six stages of the UPG model.

1. Transfer of productive assets

Asset transfer is ideal for those CHVs who desire to run their own business. This is what the CHVs in this study preferred could be offered in the form of cash or in-kind.

“Apart from giving us money there are things that we can benefit from… If he [benefactor] gives us dairy cattle individually, that gives me milk…. that will benefit me…” (R3, FGD 6, Kaloleni).

The KIIs likewise agreed, with one stating that the benefit would serve the community as a whole.

“…I can also think of…a boda-boda [motor bike]… used for referrals to the facility…that will boost health…if they have their own they will maybe charge less” (KII 02).

CHVs would appreciate some asset transference in the form of a tangible asset as capital, e.g. a motor bike to ferry passengers for a fee or in the form of poultry or livestock from which they could increase their earnings. Although this is the most capital intensive aspect of the programme, previous studies showed that an increase in the total asset-value index led to a positive impact on poverty alleviation [35]. The sustainability of this core component of the UPG model over time has, however, been criticised [36]. Seen through the lens of risk and vulnerability, the provision of tangible productive and household assets is considered sustainable when firstly, a minimum acceptable level of consumption is provided, secondly, protecting those considered vulnerable are protected from shocks and adverse events that would require them to sell their assets and thirdly, this vulnerability is prevented in the long-term [37]. The policy implication includes determining the threshold for “acceptable” consumption level vis-à-vis how sustainable this is.

2. Weekly stipends/consumption support

Food security is a key concern and may hinder CHVs from taking risks on long-term livelihood activities. Providing a safety net that meets the basic needs of the CHVs such as a monetary compensation for the time they provide their services will allow them to engage better in programme activities as well as enable them to focus on building self-sustaining livelihoods. The CHVs in this study requested for allowances and other support to conduct their duties. This was echoed by the KIIs.

“..[As] has been said….is that token… be increased….they should look at what they will give the CHVs so that they can be well motivated.” (R3, FGD 5 Kaloleni).

“…if there could be some arrangement that they get some payment… I would support that…at least something that can make someone to feel that at the end of the month… he can move on.” (KII 01).

“….what can give us the morale is they think about us at least every month…someone will know even if I lose that way [lost opportunity to earn while volunteering], when a certain day reaches I will get something.” (R4, FGD 3, Kaloleni).

The need for financial incentives was reiterated repeatedly. For the CHVs in this study, it appeared that a fallback plan provided regularly such as a wage/salary would cushion them on the days they forfeited personal businesses to conduct their CHV-related activities. The aim of consumption support through weekly stipends in the UPG model is to ensure stabilisation of consumption and deter the risk of sale of productive assets for immediate consumption needs [27]^28^. It is thus provided until the asset begins to yield an income [27]. It is a relatively simple process and less labour intensive, however, concern arises when the stipend amounts are standardised rather than customised based on household needs creating an issue with equity [26]. Estimating consumption needs and justifying distribution based on these needs are among the policy implication aspects of this incentive.

3. Intensive hands-on training

The CHVs requested technical and vocational training to equip them with skills required for self-employment. Training is tailored to their unique needs and structured to link to a specific livelihood activity. The CHVs in this study requested for enhanced training in the informal sector, in entrepreneurship and best practice in agricultural farming and livestock rearing.

“…We need…training about rearing [livestock] so, that we can know how to keep chicken…and also those drugs that are used when chicken have been affected by any disease and the feeds.” (R7, FGD 9 Kaloleni).

“… Projects…require good management…if it would be possible to help us in bringing leadership training so that we… [are] able to manage those projects that we are even given without difficulty so that we can succeed.” (R1, FGD 2 Rabai).

The KIIs participants also felt that training was important. They suggested that it should accompany the health education training provided for the CHVs to perform their community health activities.

“…if they are taught about health, they should also have trainings teaching how to start their own businesses.” (KII 04).

Additionally, some businesses were not productive because basic training on running core businesses was lacking.

“..Of their dairy cattle… but they are not producing simply because they haven't been held their hands and told this and that should be done.” (KII 03).

Responses from the CHVs pointed to technical or entrepreneurship enhancement that most felt would enable them improve their financial capacity and in this way empower them economically. The hands-on training within the UPG model is designed to provide transferable skills that will enable users to maximise the income-generating asset they are provided [27]. Provided before and after provision of the assets, beneficiaries are usually able to outpace their peers in per-capita income based on the training they receive [26]. Additionally, they also benefit from increased access to new labour markets and unlocking access to new job opportunities [35]. Hands-on training, however, requires significant human resource, more supervision, is time-intensive and requires adaptability to the local context [26]^36^. In resource-poor settings, this can be a huge limitation.

4. Savings

Learning to save one’s income and resources is an integral tool and doing so consistently helps instil a saving culture while expanding ones’ assets. The CHVs in this study expressed desire to save, including a need for education on how to save. The KIIs participants also shared these sentiments.

“…Help us…. save money that can be capital…….so [we] can do something and return with interest…that money can sustain…and the group continues.” (R8, FGD 10 Kaloleni).

“…Give them [CHVs] some allowance….then out of this allowance they will be able to save something and….appreciate…and [be encouraged].” (KII 08).

Building up their savings pool was seen as a way of enhancing economic freedom. The cash would subsequently be ploughed into their businesses to grow their investments. Saving groups were the preferred option as they promote wealth accumulation and boost household resilience [36]. Additionally, when pooled, the savings may be used for joint business developmental projects just as proposed by the CHVs in this study. Over time however, from the studies that have used the UPG model, this has had negative results with participants saving less towards the end of the intervention [26]. The likely reason for this is the elastic relationship between increasing income and savings, whereby, possibly as a result of business expansion, borrowing and the need to save decreases [27].

5. Healthcare

CHVs are community champions of health yet they had limited access to healthcare services. The financial burden of meeting their own healthcare needs was also enormous. They expressed desire for direct access to healthcare services including tokens for themselves and their family members, thereby enabling them to focus on the healthcare needs of their fellow community members.

“My request is for our hospital, XXX, they should stock the drugs for us because there are those who can go to the chemist and there are those who cannot….Our people suffer… She was taken there… told there is no medicine she has to go and buy, she returned and stayed at home with her illness.” (R4, FGD 2 Rabai).

The CHVs believed that they at the very least deserve access to basic/essential medical services at no additional cost. They were sometimes unable to afford even cheap medications. Some left medical facilities unattended to due to lack of finances. The UPG model recognises health support through two strategies, the first being education and information dissemination, the second being healthcare provision [27]. However, sustainability is a concern. Physical infrastructure such as water and sanitation facilities, and healthcare infrastructure and resource where limited, impede sustained progress at the household levels particularly where UPG intervention beneficiaries remain vulnerable to macro-level and ecological shocks [26].

6. Social integration

The CHVs in this study emphasised a consistent thread of a need for community engagement, acceptability and identity. CHVs are the social link between communities and the healthcare system. A lack of recognition by and integration into the community is a major challenge at meeting their programme-related duties. The CHVs in this study requested that they get some form of recognition provided by the government or CHV programmes within the community. They also asked for formal introduction to the community leaders and consideration for engagement or inclusion during governance meetings that concern the communities they serve.

“…If we…can be recognized…we can get a badge, t-shirt so when we get in the community they know these are the CHVs,….there are places [where] we are despised,…we go just with our clothes…if you tell them to dig a toilet they tell you go tell the doctor who sent you to come and dig that toilet…but if we have the apparels…they will give us the respect and even our work will continue on well”. (R5, FGD 2 Rabai).

“We would like to have uniform…bags…, then people will respect us, they will say these women are working at the hospital.” (R7, FGD 6, Kaloleni).

On social integration, the KIIs had this to say:

“…If we gave them space in our facility… [then] they can have a central place where they can come and discuss their things with us.” (KII 06).

In this way, they are suggesting some shared space and partnership with the CHVs in form of community embeddedness. This is the final stage of the UPG model by which time, through the model the social capital, social wealth and developments of beneficiaries is believed to have grown through the intervention [27]. This creates a sense of social prestige [26]. In our study, the desire for community embeddedness was similar to other studies where CHVs reported that recognition by their communities built social capital for them, strengthening their motivation to continue working and increased their accountability to the communities they served [3] 38. In addition, self-worth is cultivated among individuals due to improved visibility and empowerment within their communities, providing a sense of control over their livelihoods. Interventions such as the UPG model in this regard has social and economic policy inclusivity implications whereby the economic empowerment it provides gives the poor an opportunity to voice their needs and reduces discrimination [35].

## Discussion

CHVs tend to work in environments where there is limited access to formal health services and in communities that are generally poor [39]. CHVs are themselves poor, thereby needing and expecting an income. Although not inexpensive, with the right political goodwill, an investment in CHV programmes where consistent financial, technical and material support are provided have demonstrated lasting effect and impact [2]^39^. A successful intervention programme requires an understanding of the needs of the target population and tailoring the model to meet these needs. This current study delivers important findings as it provides a user-defined approach to identify and assess the needs of CHVs for socio-economic empowerment.

In the present study, the application of the UPG model appeared to mirror quite well the variety of ways in which CHV felt they could be motivated to remain engaged in their roles. Foremost is as to whether CHVs should remain as volunteers while engaged in this important role, an issue that remains controversial. Some studies suggest that CHVs should volunteer willingly without expectation of monetary gain [18]. Despite this, previous studies have demonstrated that financial and non-financial incentives are ways in which programmes can enhance retention of CHVs [38]. In this study, each of the six components of the UPG model were aspects found to be of import to the enhanced motivation of CHVs. While the model improves the lives of the ultra-poor along various dimensions, it is not without limitations. Graduation programmes are expensive and therefore their cost-effectiveness is yet to be addressed [36]. Additionally, the impact of the intervention over longer periods of time are yet to be explored, as is the viability of transformative impact through training which in order to operate at scale requires quality training, hiring and supervision [28]. The impact of these kinds of models depend therefore on scale, coverage, targeting and implementation strategies alongside social protection policies [35]. The graduation model focuses on the individual however; other factors beyond the programme may influence its outcomes. These include constraining household-specific characteristics such as women-headed or male-headed households, lack of adequate physical and health infrastructure, macroeconomic shocks such as global financial or fuel crises and an absence of market in severely deprived areas [26].

The UPG model, as described in this study proposes an opportunity for an integrated social protection intervention with broad macroeconomic impact. Investing is integrated social protection interventions and can achieve improvements in multidimensional deprivations such as education, health and nutrition and address several of the Sustainable Development Goals simultaneously [40]. Previous research on social protection is focused on wealthier countries and whether these programmes will realise similar success in low–middle income countries requires further research [41]. Sustainable success of poverty alleviation using the graduation approach is dependent on the presence of support services that reinforce the pathway of households from poverty using a combination of asset financing, mainstream development programmes and government driven social protection programmes [40]^4243^.

### Strengths and limitations of this study

A key strength of this study is that it uniquely captures the voices of the CHVs and key stakeholders and applies the UPG model to synthesise findings and contextualise them in the local setting. Given that this work was embedded in a larger programme, our findings may over-represent a “motivated” sample who receive certain allowances such as travel allowance, training and project support to be able to engage in their activities. Lastly, it is likely that due to their affiliation with the programme there may have been some social desirability bias in the responses provided by the CHVs.

## Conclusion

The results of this study suggest that there exists opportunity for a growth trajectory that can sustainably provide economic empowerment of the CHVs working in Kenya. A platform that provides social assistance harnessing the key tenets of the Ultra-Poverty Graduation Model, where seed capital is used to jumpstart an economic activity combined with sufficient financial access and education on management potentially provides this opportunity. For this to be successful in Kenya, it would require both local government and multi-sectoral collaboration, as these endeavours are both capital and time intensive.

## Supplementary Information

**Additional file 1: Appendix 1.** Study information and informed consent form. **Appendix 2.** CHVs Focus Group Discussion Interview Guide. **Appendix 3.** CHVs Key Informant Interview Guide.

## Data Availability

Transcripts from this study cannot be shared publicly due to ethical consideration. Researchers meeting the criteria for access to confidential data can contact the following individuals njeri.nyanja@aku.edu OR anthony.ngugi@aku.edu.

## Abbreviations

AQCESS: Access to Quality Care through Extending and Strengthening Health Systems
BRAC: Bangladesh Rural Advancement Committee
CBO: Community-Based Organization
CHU: Community Health Unit
CHV: Community health volunteer
CSO: Civil Society Organization
FGD: Focus group discussion
IGA: Income generating activity
KII: Key Informant Interviews
LMICs: Low- and middle-income countries
MOA: Ministry of Agriculture
MOH: Ministry of Health
PI: Principal investigator
RMNCH: Reproductive Maternal Neonatal and Child Health
UPG: Ultra-poverty graduation
